# First dorsal metacarpal artery islanded flap: A useful flap for reconstruction of thumb pulp defects

**DOI:** 10.4103/0970-0358.53003

**Published:** 2009

**Authors:** Chetan Satish, Sunit Nema

**Affiliations:** Department of Plastic Surgery, K.E.M Hospital, Mumbai - 400 012, India

**Keywords:** First dorsal metacarpal artery flap, Thumb pulp defect, Sensate flap

## Abstract

Thumb pulp defects are commonly due to avulsion injuries. It is very important to reconstruct these defects using sensate flaps as the thumb pulp needs to be sensate for implementing the various functions of the thumb. A very good option for coverage of these defects is the islanded first dorsal metacarpal artery flap. Our study was done over a period of 2 years and involved 9 consecutive cases of thumb pulp defects treated at our institution. The patients included 8 males and 1 female, ranging in age from 16 to 51 years old. The flap size ranged from 2 × 1.5 cm to 5 × 3 cm. We had only one complication in the form of partial flap necrosis, which fortunately healed following debridement without the need for a secondary procedure. All our cases were done under local anesthesia with tourniquet control. All the patients had good fine touch and average two-point discrimination of 6 mm, which was satisfactory. Our good results further reinforce the islanded first dorsal metacarpal artery flap as one the best flaps for sensate reconstruction of thumb pulp defects. It replaces the soft tissue loss at the thumb pulp with minimal donor site morbidity and with good return of thumb pulp sensation.

## INTRODUCTION

Thumb pulp defects are commonly due to avulsion injuries. While superficial defects can be treated with skin grafts, deeper defects with exposure of tendon or bone are more difficult to reconstruct. It is very important to reconstruct these defects using sensate flaps as the thumb pulp needs to be sensate for implementing the various functions of the thumb.

Various options used for reconstruction of thumb pulp defects include Littler's neurovascular island flap,[1] pulp tissue transfer of toe and other small free flaps,[2] and sensate cross-fingered flaps.[3] A very good option for coverage of these defects is the islanded first dorsal metacarpal artery flap.[4–6]

The first dorsal metacarpal artery flap (FDMA) was first described by Foucher and Braun who demonstrated that a sensate skin island flap created from the dorsum of the index finger could be raised based upon the first dorsal metacarpal artery and sensory branch of the radial nerve.[7]

The first dorsal metacarpal artery is a constant vessel arising from the radial artery just distal to the tendon of the extensor pollicis longus and proximal to the point at which the radial artery pierces between the radial and ulnar heads of the first dorsal interosseous muscle. The artery runs over the fascial layer of the first dorsal interosseous muscle and divides into the radial branch to the thumb, the intermediate branch to the first web space, and the ulnar branch to the index finger.[8]

## AIMS AND OBJECTIVES

The aim of this study was to evaluate the usefulness of the islanded first dorsal metacarpal artery flap for coverage of thumb pulp defects and to review the literature for reconstruction of thumb pulp defects.

## MATERIAL AND METHODS

Our study was done over a period of 2 years and involved 9 consecutive cases of thumb pulp defects. The patients included 8 males and 1 female, ranging in age from 16 to 51 years. All the patients had avulsion injuries and the patients were treated as emergency cases. All the flaps were studied for sensory return in the form of fine touch and two point discrimination.

### Surgical Technique

All our cases were done in the emergency room under local anesthesia with tourniquet control. The flap size determined by the defect of the thumb following debridement was made over the proximal phalanx of the adjacent index finger. The width of the flap was so designed that it does not extend beyond the radial and ulnar midaxial lines of the proximal phalanx. The flap is raised from the distal to the proximal side and from the ulnar to radial side. The tourniquet was released following flap harvest to ensure viability of the flap. The tunneling, flap insetting, and grafting of the donor area were done without tourniquet control.

After preoperative planning and markings, the limb was exsanguinated and the tourniquet was raised. The flap must not extend beyond the proximal interphalangeal joint[5] although the extended first dorsal metacarpal artery island flap including the skin over middle phalanx has been described.[6]

**Figure 1:**
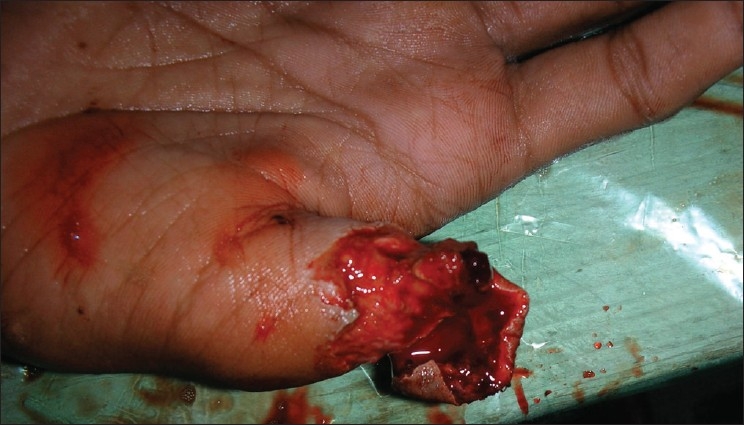
Thumb pulp defect

**Figure 2:**
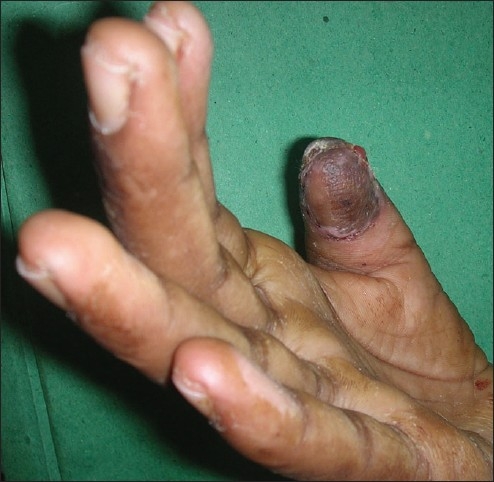
The FDMA flap inset into the thumb pulp defect

**Figure 3:**
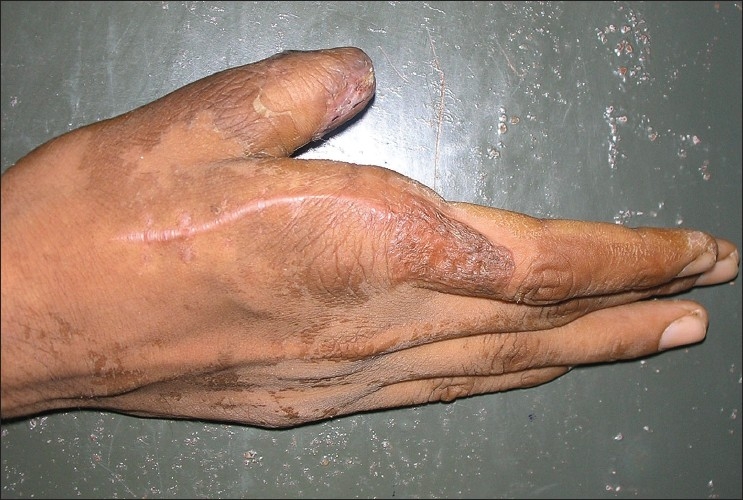
The grafted donor area

The skin on the dorsum of the hand proximal to the flap was first incised in a zigzag manner and undermined to identify the first dorsal metacarpal artery and its branch to the index finger. Sensory branches to the index finger were identified. The plane for undermining was sub dermal to avoid injury to the pedicle in the subcutaneous tissue. After identifying the main pedicle, the branches were cauterized using bipolar cauterization. The flap was then harvested distally taking a sufficient cuff of subcutaneous tissue along with the pedicle proximally. After ensuring sufficient pedicle length, the flap was tunneled into the defect, which had been sufficiently debrided [Figure 1]. The islanded flap was insetted after ensuring adequate bleeding [Figure 2]. Care must be taken to preserve the paratenon at the donor area of the flap. The donor area was covered with a split thickness graft harvested from the thigh area under local anesthesia [Figure 3]. Graft dressing and protective splinting were applied.

**Figures 4 and 5:**
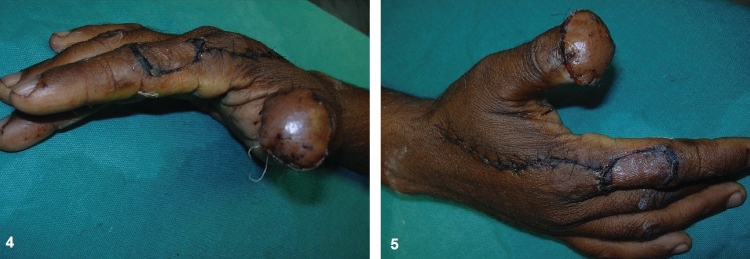
The flap and its donor area

The graft dressing was done on the 5^th^ day. The hand and fingers were mobilized once the graft settled usually at 2 weeks [Figures 4 and 5]. Post-operatively, the patient was evaluated for sensory return with tests for fine touch and two-point discrimination.

## RESULTS

The clinical data is summarized in Table 1. The flap size ranged from 2 × 1.5 cm to 5 × 3 cm. We had only one complication in the form of partial flap necrosis, which fortunately healed following debridement without the need for a secondary procedure. All our cases were done under local anesthesia with tourniquet control. The tourniquet was used only until the flap was harvested, which all patients could tolerate. After releasing the tourniquet, flap bleed was ensured and the rest of the procedure was done without a tourniquet. The average duration of surgery was 45 minutes, whereas the longest duration of surgery was 1 hour 10 minutes. The majority of the cases were done under the naked eye with a surgical loupe being used only in cases in which there was difficulty in visualizing the pedicle. All the patients had good fine touch and average two-point discrimination of 6 mm, which was satisfactory. One peculiar problem encountered was the feeling of sensation of the flap coming from the dorsum of the index finger, which the patient's learnt to adjust to over time (double sensibility phenomenon).[1] Cortical disorientation did not persist and faded with time.

**Table 1:** Clinical data

*Gender/Age*	*Flap size (cm)*	*Complication*	*Static 2-PD (mm)*
M/20	3 × 1.5	Nil	6
M/24	2 × 1.5	Nil	6
M/51	4 × 2.5	Partial necrosis	7
M/28	5 × 3	Nil	7
M/16	3 × 2	Nil	6
M/27	3 × 1.5	Nil	6
F/22	3 × 2	Nil	6
M/34	4 × 2	Nil	6
M/30	3 × 1.5	Nil	6

M = Male; F = Female; 2-PD = 2-point discrimination.

Thumb length is usually less than the level of proximal interphalangeal joint of the index finger, and since we have used an islanded flap; its reach to the tip of the thumb was never a problem. In case such a problem arises, the extended first dorsal metacarpal artery flap as described in the literature can always be used.[6]

## DISCUSSION

Historically, thumb pulp defects were initially reconstructed using non sensate flaps such as the cross-fingered flaps. However, the realization of the importance of a sensate pulp led to the use of sensate flaps.

Littler's neurovascular island flap is one such useful flap. However, the incorporation of the digital vessel is a major disadvantage.[1] Other alternatives are the sensate cross-fingered flap, which is quite tedious as it requires micro vascular co-aptation of the nerves.[3] Small free flaps such as the toe wrap around flap are alternatives that we believe are overkill compared with the simplicity of the harvest of the islanded first dorsal metacarpal artery flap.

The pedicle of the first dorsal metacarpal artery flap with an average length of 7 cm includes the ulnar branch of the first dorsal metacarpal artery, the dorsal veins, and the cutaneous branch of the radial nerve.[9,10]

Although the first dorsal metacarpal artery flap and its applied vascular anatomy have been well documented in literature, very few articles have focused on its usefulness for reconstruction of thumb pulp defects.[4]

Its advantages include the ease of harvest- it does not require much expertise and can be performed under local anaesthesia. Also, It is a sensate flap with minimal donor site morbidity.

Its disadvantages are the size limitation of the flap, which can extend distally to the proximal interphalangeal joint and proximally to the metacarpophalangeal joint, and possible donor site graft loss if paratenoon is not preserved.

## CONCLUSION

Our good results further reinforce the island first dorsal metacarpal artery flap as one the best flaps for sensate reconstruction of thumb pulp defects. It replaces the soft tissue loss at the thumb pulp with minimal donor site morbidity and with good return of thumb pulp sensation.

Thumb pulp defects with tendon and bone exposure are not so common. Even though in our series only 9 cases have been reported, the purpose of this article is to stress the extreme ease with which this flap can be harvested with minimal donor morbidity and its success in restoring a sensate pulp.

The use of this flap must be explained to the junior hand surgeons as they are often the ones treating thumb pulp defects in the emergency.

We hope this article will reach out to the junior hand surgeons so that this useful flap can be utilized more effectively. This is the primary aim of this article.
